# Biosynthesis gene cluster‐containing species drive microbial community stability under thermal stress through enhanced ecological cohesion

**DOI:** 10.1002/imo2.70082

**Published:** 2026-02-15

**Authors:** Shibo Ban, Binghao He, Yang Song, Xin Li An, Yan Xu, Qun Wu, Yu Shi

**Affiliations:** ^1^ Key Laboratory of Industrial Biotechnology of Ministry of Education, State Key Laboratory of Food Science and Resources, School of Biotechnology Jiangnan University Wuxi China; ^2^ Kweichow Moutai Co, Ltd Zunyi China; ^3^ Guizhou Key Laboratory of Microbial Resources Exploration in Fermentation Industry Kweichow Moutai Group Zunyi China; ^4^ Plant‐Microbe Interactions, Institute of Environmental Biology, Department of Biology, Science4Life Utrecht University Utrecht the Netherlands; ^5^ State Key Laboratory of Crop Stress Adaptation and Improvement, School of Life Sciences Henan University Kaifeng China

**Keywords:** biosynthesis gene clusters, ecological cohesion, fundamental species, microbial community stability, thermal stress

## Abstract

Understanding the mechanisms that govern the ecological stability of microbial communities is critical to elucidating how microbes adapt to changes in their environment. Here, we investigated microbial assembly and changes during spontaneous fermentation, in which the temperature naturally increased from 20°C to 65°C, to determine how thermal stress shapes community dynamics and resilience. Longitudinal metagenomic sampling revealed that heterogeneous selection favored thermotolerant taxa, while ecological drift exerted a stronger influence on fungal communities, indicating that both deterministic and stochastic processes jointly contributed to the community assembly. Specifically, bacterial turnover was primarily driven by heterogeneous selection and dispersal limitation, while fungal assembly was driven by homogeneous selection and drift. Furthermore, the infer Community Assembly Mechanisms by Phylogenetic‐bin‐based null model analysis identified 27 bacterial and 140 fungal groups that exhibited significant compositional shifts along the thermal gradient. Among these, 11 biosynthesis gene cluster (BGC)‐containing species could enhance the microbial community stability by strengthening positive cohesion among species (all species: 0.303–0.464; without BGC‐containing species: 0.289–0.461). By removal simulations, we further identified six BGC‐containing species (two bacteria and four fungi) that were critical for maintaining robustness to the changes under thermal stress. Co‐culture experiments confirmed that their presence increased community biomass by at least 50.08% compared with the control without these six species. Taken together, our results suggest that BGC‐containing species can maintain the microbial community stability by reinforcing ecological cohesion and increasing biomass production under high‐temperature stress.

## Highlights

- The iCAMP phylogenetic‐bin analysis revealed stage‐specific shifts in community assembly processes along the natural *Daqu* temperature trajectory (20°C–65°C).
- Biosynthesis gene cluster (BGC)‐containing phylogenetic bins and fundamental species were associated with higher ecological cohesion and stability‐related network properties under thermal stress.
- Molecular ecological network analyses across temperature intervals identified key taxa whose removal disproportionately reduced network robustness and collapse threshold.
- Isolate‐based SynCom experiments showed temperature‐dependent biomass differences across inoculation ratios of fundamental species versus non‐BGC species, supporting their functional relevance during heating.

## Graphical abstract

The study framework for exploring microbial community assembly and community stability of biosynthesis gene cluster (BGC)‐containing species under thermal stress (20°C–65°C) during 39 days of spontaneous fermentation. Temperature gradients during spontaneous fermentation reshape microbial community assembly, driving pronounced succession in both bacterial and fungal communities. Community assembly is jointly governed by deterministic and stochastic processes, with bacterial heterogeneity selection and dispersal limitation showing strong associations with thermal stress, while fungal assembly is primarily linked to homogeneity selection and drift. In parallel, BGC‐containing species are enriched at high temperatures and are associated with higher community cohesion and stability‐related network properties under thermal stress. Removal simulations identified key fundamental species, indicating their importance for community resilience, while co‐culture experiments demonstrated that BGC‐containing species significantly increased biomass under thermal stress. These results underscore the critical role of BGC species in promoting stability and resilience.

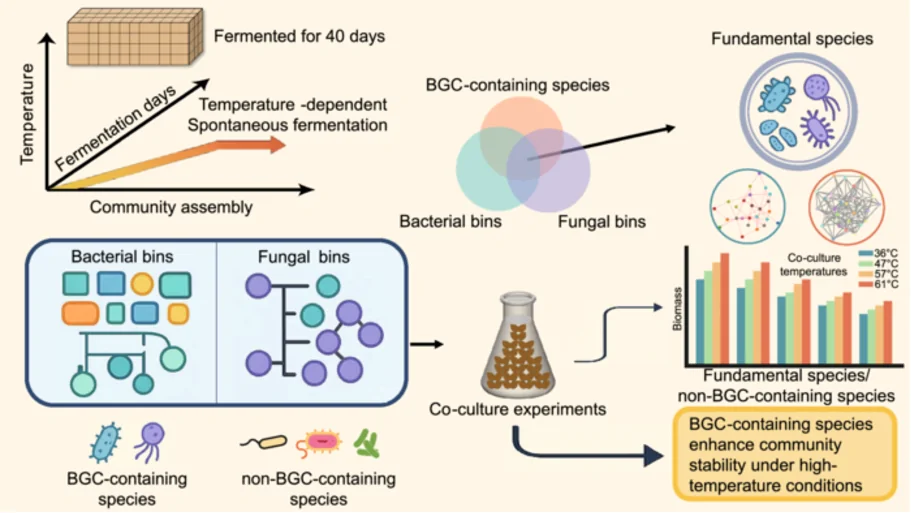

## 1 INTRODUCTION

Microbial communities are central to ecosystem functioning. However, their stability is increasingly challenged by climate change, especially rising global temperatures [1, 2, 3]. In microbial ecosystems, deterministic factors such as environmental conditions, niche differentiation, heterogeneity of nutritional resources, and interspecies interactions have proven insufficient to fully understand community dynamics. While diversity indices and similarity metrics have been used to study these patterns, they cannot fully disentangle the relative roles of deterministic versus stochastic forces [4, 5]. The community assembly process reflects both the interactions among communities and the effect of external environmental factors, and phylogenetic groups (bins) play crucial roles in microbial community assembly. Temperature gradients profoundly regulate microbial growth, succession, and ecosystem functions [6, 7, 8]. Faced with the challenge of rising temperatures, microbial communities exhibit diverse strategies for adaptation [9, 10, 11, 12]. However, the temperature‐responsive reconfiguration of assembly mechanisms and their role in sustaining stability remain unresolved [13, 14]; understanding these mechanisms is crucial for predicting resilience under warming.

During microbial community assembly, certain key functional gene units (referred to as bins) play pivotal roles in shaping assembly ecological structure and functional stability [15]. Community assembly is governed by five processes: homogeneous selection (HoS), defined as deterministic filtering that drives communities to converge under similar environments; heterogeneous selection (HeS), referring to environmental differences that cause divergence; dispersal limitation (DL), which occurs when restricted movement prevents taxa from reaching suitable sites; homogenizing dispersal (HD), in which strong dispersal mixes communities and increases similarity; and drift (DR), reflecting random birth‐death fluctuations in the absence of strong deterministic forces. Temperature profoundly regulates microbial growth, succession, and ecosystem function [16], yet how temperature gradients restructure assembly mechanisms and support stability remains unclear.

Microorganisms adapt to high‐temperature environments by modifying their cell membrane composition [17], upregulating heat shock proteins [18], and adjusting metabolic/genomic pathways [19, 20, 21], shifting dominance toward thermotolerant taxa. However, the ecological roles of key bins under temperature‐driven selection are poorly characterized. Beyond assembly processes alone, microbial genomes encode functional traits that can act as anchors of stability. Among these, bins harboring biosynthesis gene clusters (BGCs) are functional hotspots mediating chemical interactions essential for stress response and interspecies cohesion. Under temperature stress, microorganisms activate specific BGCs of secondary metabolites through epigenetic regulation and signal transduction pathways [22], synthesizing antibiotics [23, 24] and bioactive compounds [25, 26] to increase environmental adaptability, which demonstrate potential for biotechnological applications. For instance, elevated soil temperature increases resistance in antibiotic‐resistant bacteria and alters gut microbiota composition of giant African snails [23]. Community composition also modulates secondary metabolism in multispecies consortia [27]. Although bins and BGCs act synergistically under heat stress, their interaction networks during dynamic temperature fluctuations remain largely uncharacterized. Spontaneous fermentation offers a natural model to study rapid temperature increases. Influenced by ambient conditions [28, 29], it spans a broad temperature range and promotes enrichment of fermentative species adapted to anaerobic environments, yielding metabolically competent and resilient communities [30]. However, when these key species drop below a threshold abundance, functional stability can be lost, triggering regime shifts. Integrating abundance and diversity patterns reveals how these dynamics shape community structure and function, underscoring microbial adaptability to rapid environmental change [31, 32].

In this study, we examined the spontaneous fermentation, which allowed rapid natural temperature increases. The spontaneous fermentation system is *Daqu*, which is the starter used for Chinese liquor production. Raw material (wheat) was smashed with water, and the mixture was pressed as bricks (37 cm × 28 cm × 7 cm) and piled up into five layers in a fermentation room (6 m × 3.8 m × 4.0 m) for 39 days of fermentation. Through the utilization of cereal grains (primarily wheat) and mediation by spontaneously fermented starters, solid‐state fermentation generates steep thermal gradients, rising from 20°C to 65°C, and thus provides an excellent model for high‐temperature natural systems [33]. High‐temperature fermentation represents a typical process of natural heating and accumulation, serving as an excellent model for high‐temperature natural systems. As a relatively simple yet closed microbial ecosystem, fermented foods offer a compelling model for in situ testing and validating fundamental microbial ecological principles. The integration of metagenomics and metabolomics enables fermented food systems to be dissected via complex metabolic and network‐based models, which now support the development of predictive, machine‐learning‐based functional tools. Given its unique ecological characteristics, fermentation is considered an ideal biosystem for demonstrating how microbial interactions react to temperature stress. In this study, we continuously monitored microbial communities and abiotic stress with the metagenome during fermentation. We hypothesize that (1) the stability of microbial communities under temperature stress depends on a subset of fundamental species, such that a drop in relative abundance of these species below a critical threshold leads to a loss of resilience and a drastic shift in community structure and function; and (2) the presence of these fundamental species is associated with higher ecological cohesion and stability‐related network properties under thermal stress, potentially mediated by BGC‐encoded secondary metabolism.

## 2 RESULTS

### Influence of time and temperature on microbial assembly processes

To disentangle the effects of fermentation time and temperature on microbial assembly, we monitored starter fermentation over 39 days as the temperature rose from 25.5°C to 65°C (Figure S1). Eighty‐three samples were collected at seven time points (*n* = 3 each). Metagenomic sequencing yielded 440.19 Gb of raw data; 324.12 Gb clean reads were assembled into 22.27 Gb contigs, with >99% estimated coverage and 13.88 million nonredundant genes. Twenty‐nine genera with Reads Per Kilobase Million (RPKM) > 20 were dominant (Figure S2). Nonmetric multidimensional scaling (NMDS) showed no significant differences over time (Analysis of Similarities (ANOSIM): *R* = 0.040, *p* = 0.066) but significant differences across temperatures (*R* = 0.106, *p* = 0.007). α‐diversity also varied with temperature (*p* < 0.05) but not with time (Figure 1A,B and Table S1).

**FIGURE 1:**
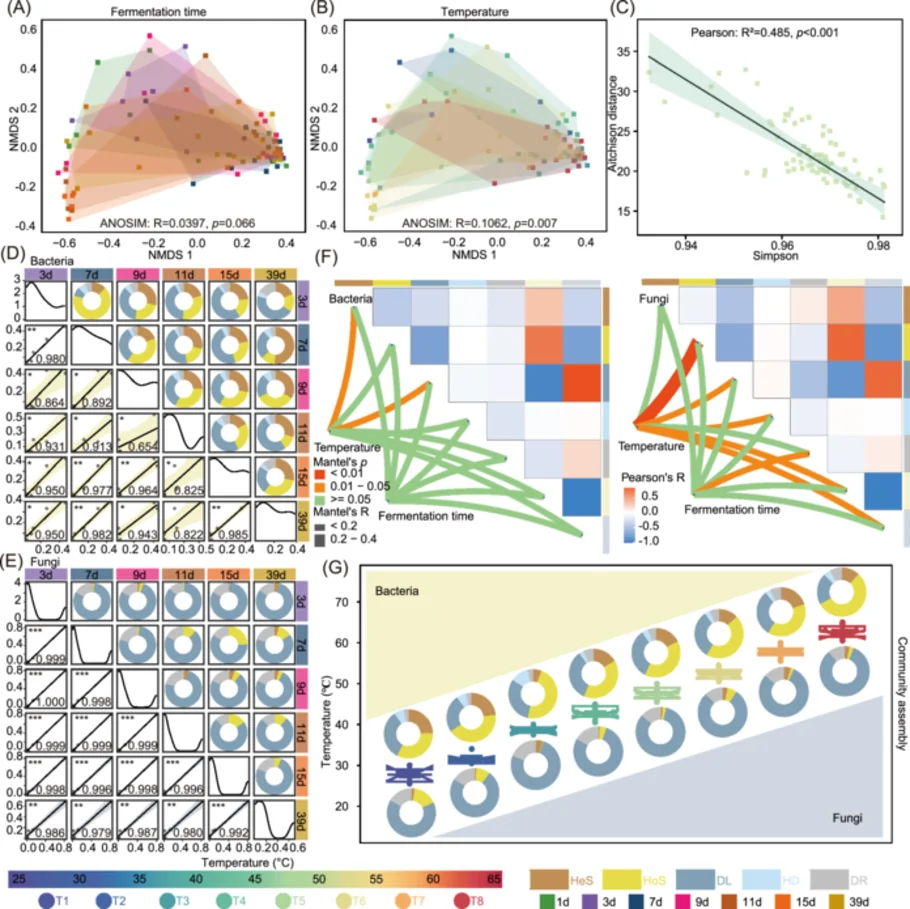
Variations in community assembly processes at different fermentation times and temperatures. Nonmetric multidimensional scaling (NMDS) for the microbial community to analyze the effects of fermentation time (A) and temperature (B) on microbial communities as analyzed with ANOSIM; (C) the correlation between β‐distance (Aitchison distance) and the Simpson index, each point represents one pairwise comparison; β‐distance is the Aitchison distance between two communities, the corresponding Simpson index represents the average α‐diversity of that pair. Aitchison distance representation, which is based on the proportional distance between samples, based on the Euclidean distance of the centered log ratio transform (CLR) vector, was used to compare metric differences in microbial structure and composition between two microbial communities. Each β‐distance was calculated as the pairwise distance between all communities at different temperatures; the relative importance of the bacterial (D) and fungal (E) assembly processes at different times was determined; each matrix square represents the estimated contribution of that assembly process between two sampling days, and the accompanying scatterplots show the regression between process contribution and fermentation day. All iCAMP analyses were performed using taxonomic abundance data. The regression equation, Pearson R value, and significance markers are located at the bottom left, whereas the community assembly process at different time points is shown at the top right. (F) The correlation between temperature/fermentation time and community assembly processes. The width and color of the lines indicate the Mantel *R* value and Mantel *p*‐value, respectively. Lines with the Mantel *p* < 0.05 indicate significant correlations, and Mantel tests were conducted using temperature/time matrices as environmental variables and iCAMP‐derived process fractions as community matrices. (G) Community assembly processes in response to temperature variation. Box plots illustrating the distribution of sample counts across 8 temperature gradients. Samples were reordered by their observed fermentation temperature, and each box plot summarizes the proportions of the five processes within the corresponding 5°C interval (T1–T8). DL, dispersal limitation; DR, drift and others; HeS, heterogeneous selection; HoS, homogeneous selection; HD, homogenizing dispersal. T1: 25°C–30°C, T2: 30°C–35°C, T3: 35°C–40°C, T4: 40°C–45°C, T5: 45°C–50°C, T6: 50°C–55°C, T7: 55°C–60°C, T8: 60°C–65°C. ***p < 0.001, **p < 0.01, *p < 0.05. Colors for fermentation days (1–39 d) are consistent across (A), (D), and (E); colors for temperature bins (T1–T8) are consistent across (B) and (G).

Given the minimal impact of fermentation time on microbial community structure, we next investigated alternative drivers of community assembly dynamics. A significant correlation was observed between α‐diversity and β‐diversity (Aitchison distance) (Figures 1C and S3; *R*² = 0.485, *p* < 0.001), indicating that despite the stable overall structure over time, internal compositional changes continued to occur. To further investigate community assembly dynamics, we applied the infer Community Assembly Mechanisms by Phylogenetic‐bin‐based null model analysis (iCAMP) to evaluate the relative contributions of different assembly processes (Table S2). The results revealed that bacterial assembly during fermentation was dominated by deterministic processes, accounting for 34.96 ± 3.90%, whereas HeS accounted for 12.92 ± 1.71% (Figure 1D). These findings suggested that temperature and microbial adaptability played dominant roles in shaping community structure. Fungal community assembly was predominantly governed by DL (78.79  ± 1.45%), indicating restricted fungal spread within the fermentation environment. In contrast, HoS (4.40 ± 1.31%) played a secondary role, whereas heterogeneous selection (HeS, 2.69 ± 0.82%) contributed minimally. These results suggested that selective pressures imposed by the fermentation environment had a limited influence on fungal community differentiation (Figure 1E).

Temperature was positively correlated with the HeS (*R* = 0.085, *p* = 0.049) and DL (*R* = 0.097, *p* = 0.042) of the bacterial community, as well as with the HoS (*R* = 0.210, *p* = 0.009), DL (*R* = 0.153, *p* = 0.018), DR (*R* = 0.075, *p* = 0.041), and both deterministic (*R* = 0.170, *p* = 0.019) and stochastic assembly (*R* = 0.170, *p* = 0.023) of the fungal community; fermentation time was positively correlated with only the DR (*R* = 0.076, *p *= 0.041) of the fungal community (Figure 1F). The partial Mantel analysis revealed that, when accounting for the influence of time, the community assembly process was significantly correlated with temperature (*R* = 0.079, *p* = 0.038). In contrast, without the influence of temperature, no significant associations were detected between the community assembly process and time (*R* = 0.027, *p* = 0.246) or acidity (*R* = −0.054, *p* = 0.903). Therefore, the importance of temperature in community assembly is much greater than that of fermentation time.

Because temperature had a significant effect on the assembly process, the changes in community assembly along the temperature gradient were analyzed (Figure 1G). DL dominated bacterial assembly, increasing at lower and intermediate temperatures (27°C–30°C) and decreasing at higher temperatures. HeS was stronger at low (25.5°C and 27°C) and high temperatures (62°C and 64°C), whereas HoS dominated in the mid‐temperature range (30°C–45°C). For fungi, HeS was generally low but higher at specific temperatures (29°C, 40°C, 58.5°C, and 64°C), whereas HoS peaked at 26°C and decreased with temperature. DL and DR were key in fungal assembly, particularly at higher temperatures (52°C–60°C). These patterns indicate that temperature, rather than fermentation time, is the primary driver of shifts in assembly mechanisms, especially under thermal stress.

### Variation in microbial communities and phylogenetic groups under high temperatures

Given the importance of temperature, we reordered the samples on the basis of temperature to identify key species at high temperatures. We then classified every 8 samples with adjacent temperatures via a stepwise clustering method to construct a molecular ecological network (MEN), and a total of 76 MENs were obtained (Figure S4). The number of observed species was almost constant (ranging from 288 to 292) among these 76 MENs (Table S3). The total microbial abundance was not significantly different across the 76 MENs (RPKM from 2030.84 to 2415.21, ANOSIM: *R* = 0.018, *p* = 0.053). These results indicated that the MEN reduced the effect of temperature on community abundance.

To characterize MENs across temperature gradients, we analyzed the relationships between temperature and key indicators, including microbiota abundance, node persistence, and Aitchison distance (Figure 2A). Node persistence, defined as the ratio of persistent nodes to visible species, reflects community stability, whereas Aitchison distance quantifies compositional differences across microbial communities. The overall community characteristics were positively correlated with temperature (Figure 2A). In addition, temperature was positively correlated with microbiota abundance (*R* = 0.399, *p* < 0.001), α‐diversity (*R* = −0.405, *p* < 0.001), β distance (*R* = 0.243, *p* = 0.036), and node persistence (*R* = 0.247, *p* = 0.017) (Figure S5). These results indicated that high temperatures drove a decline in community stability and an increase in heterogeneity through different mechanisms. The influence of different temperatures on the microbial communities was mapped to visualize their variations (Figure 2B). The community characteristics were primarily distributed across four temperature ranges: 28.06°C to 44.44°C, 44.81°C to 49.19°C, 49.87°C to 58.31°C, and 58.75°C to 63.81°C. When the temperatures ranged from 58.75°C to 63.81°C, the microbiota abundance increased from 2162.87 to 2319.34, the node persistence gradually rose again from 0.24 to 0.51, and the Aitchison distance increased from 18.56 to 32.42 (Table S3), which indicated that the increase in β‐distance was caused by the disappearance of certain nodes in the MEN.

**FIGURE 2:**
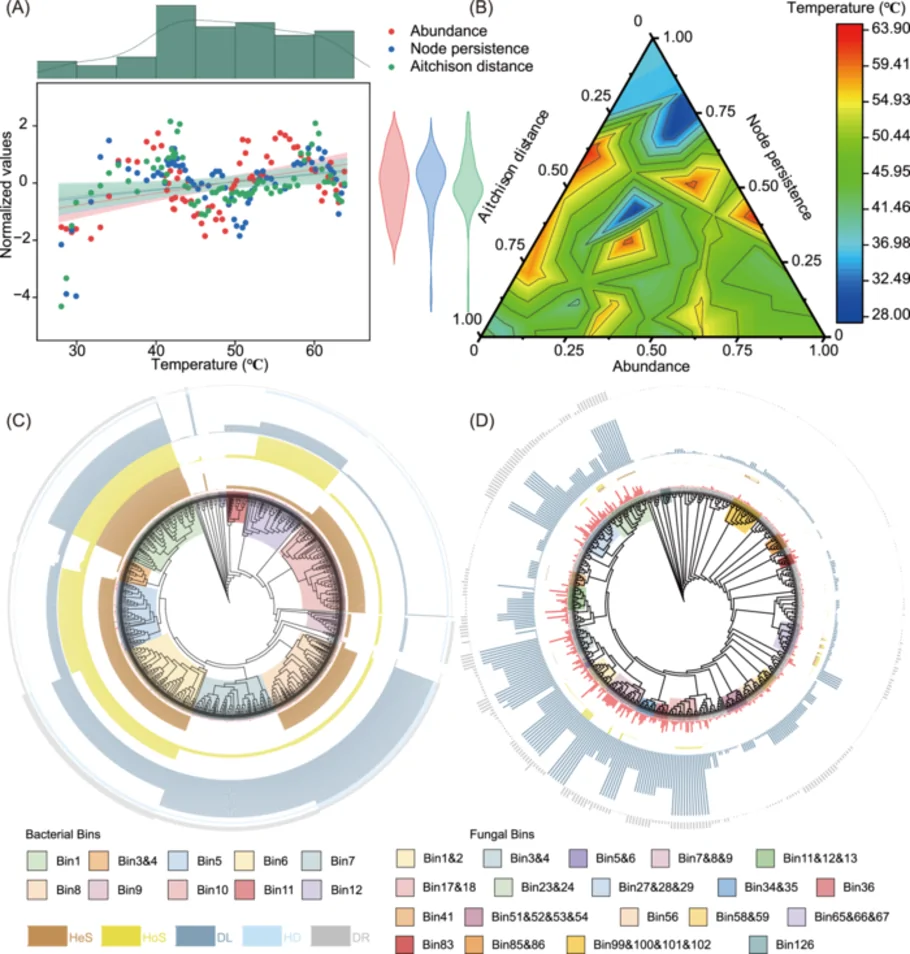
Microbial differentiation occurs during community assembly processes. (A) Normalized analysis of the β‐distance of community abundances, node persistence, and Aitchison distance with temperature changes. Aitchison distance calculated from CLR‐transformed taxonomic abundance vectors, and the bar and line overlays represent standardized values of node persistence and Aitchison distance. (B) Distribution of microbial characteristics with temperature variation, visualizing the relative magnitude of each attribute across the full temperature gradient. Phylogenetic tree (center) of bacteria (C) and fungi (D), surrounded by six concentric rings representing the relative importance of different processes: DR in the outermost ring, followed by HD, DL, HoS, and ASV abundance in the innermost ring. This illustrates the abundance‐weighted significance of these processes in each phylogenetic group on the basis of iCAMP analysis. Additionally, bins containing more than five ASVs are highlighted, and bins exceeding this threshold were merged for visualization clarity. DL, dispersal limitation; HeS, heterogeneous selection; HoS, homogeneous selection; HD, homogenizing dispersal; DR, drift and others; colors for the five assembly processes are identical in (C) and (D).

To further understand the temperature‐driven changes in microbial communities, we examined the ecological processes shaping their assembly. In the assembly processes of the observed microbial communities, 27 bins were identified for bacteria, and 140 bins were identified for fungi. For bacteria, the relative contributions of different assembly processes were as follows: HoS, HeS, DL, HD, and DR accounted for 11.11%, 11.11%, 11.11%, 14.81%, and 51.85% of the bins, respectively, corresponding to proportion of 26.84%, 24.30%, 7.35%, 4.67%, and 36.85%, respectively (Figure 2C). In contrast, for fungi, the assembly processes showed different dynamics, where HoS, HeS, DL, and DR accounted for 16.43%, 10%, 5%, and 68.57% of the bins, respectively, with corresponding relative abundances of 2.12%, 1.19%, 80.94%, and 15.70%, respectively (Figure 2D). Therefore, 27 bacteria and 56 fungi were defined as species that contribute to assembly and maintain community abundance and diversity under high temperatures. In summary, these results highlighted that different ecological processes, particularly drift, drove community assembly and maintained abundance and diversity under high temperature.

### The role of BGC‐containing phylogenetic groups in microbial community stability at high temperatures

To investigate the microbial functional groups contributing to microbial community stability at high temperatures, gene functional annotation was performed using eggNOG [34]. A total of 847,724 proteins were annotated on the basis of contigs, 242,497 proteins were associated with metabolic functions, and metabolic functions accounted for 28.7% of the total functional annotations (Figure 3A). There were 8 functional groups related to metabolic functions, and secondary metabolic function (secondary metabolite biosynthesis, transport, and catabolism) genes were significantly positively correlated with temperature (*R* = 0.230, *p* = 0.046) (Figure S6). We further analyzed the taxonomic origin of BGCs. A total of 1413 BGCs were identified across 17 dominant genera and other genera. Among these, *Paecilomyces* and *Aspergillus* emerged as the dominant genera, containing a substantial number of BGCs, particularly in the PKS (polyketide synthase) and NRPS (non‐ribosomal peptide synthetase) categories. *Paecilomyces* primarily contained the PKS category of BGCs, whereas *Aspergillus* displayed a diverse array of BGC types, including the terpene and NRPS categories. The average number of BGCs per taxon was inconsistent, with *Bacillus* showing a notable presence of both NRPS and hybrid PKS‐NRP types. More than 50% of the *Paecilomyces* and *Bacillus* containing BGCs originated at temperatures above 55°C (Figure 3B). Given their significant roles in community assembly, along with their secondary metabolic potential, in summary, we identified 173 BGC‐containing species (Table S4), and 83 community assemblies contributed bins. A total of 11 species (*Aspergillus chevalieri*, *Aspergillus nidulans*, *Bacillus velezensis*, *Enterobacter cancerogenus*, *Monascus pilosus*, *Paecilomyces variotii*, *Pediococcus acidilactici*, *Pichia kudriavzevii*, *Rhizopus microsporus*, *Staphylococcus gallinarum*, and *Weizmannia coagulans*) were designated as BGC‐containing bins (Figure 3C). To evaluate the ecological impact of specialized metabolism, we quantified the contribution of BGC‐containing bins to community assembly. Linear regression analysis identified BGC‐containing bins as the dominant drivers, explaining 80.38% of the variation. In contrast, bins containing other gene clusters accounted for far less of the variation (13.65% for bacteria, 22.97% for fungi), underscoring that BGCs were pivotal in community assembly (Figure 3D).

**FIGURE 3:**
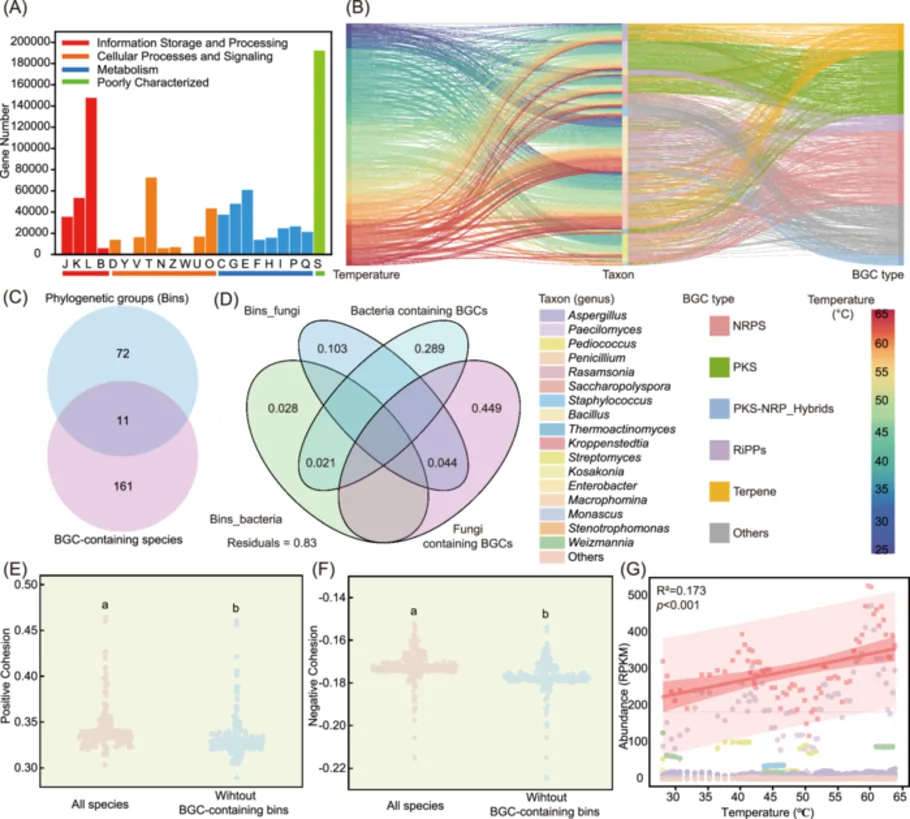
Contribution of biosynthesis gene cluster (BGC)‐containing phylogenetic groups (bins) to community stability. (A) Distribution of microbial functions based on COG categories. J, Translation, ribosomal structure and biogenesis; K, Transcription; L, Replication, recombination and repair; B, Chromatin structure and dynamics; D, Cell cycle control, cell division, chromosome partitioning; Y, Nuclear structure; V, Defense mechanisms; T, Signal transduction mechanisms; N, Cell motility; Z, Cytoskeleton; W, Extracellular structures; U, Intracellular trafficking, secretion, and vesicular transport; O, Posttranslational modification, protein turnover, chaperones; C, Energy production and conversion; G, Carbohydrate transport and metabolism; E, Amino acid transport and metabolism; F, Nucleotide transport and metabolism; H, Coenzyme transport and metabolism; I, Lipid transport and metabolism; P, Inorganic ion transport and metabolism; Q, Secondary metabolites biosynthesis, transport and catabolism; and S, Function unknown. (B) Sankey diagram showing the taxonomic origin (genus level) of BGCs at different temperatures and the composition of BGC types in different families. Species that were not included in the 17 dominant genera were combined and shown in Others. BGCs that were not included in the five dominant BGC types were combined and shown in Others. (C) Venn analysis identified 11 species that contribute to community assembly and contain BGCs. In the iCAMP analysis, the observed bacterial and fungal ASVs were divided into 27 and 140 bins, respectively, on the basis of the phylogenetic signal threshold. All BGC sequences predicted by antiSMASH were annotated, and species with at least one BGC were identified as BGC‐containing bins. Here, “bins” refer to phylogenetic clusters defined by iCAMP and are distinct from ASVs or taxonomic species. (D) Linear regression analysis was used to demonstrate the contributions of species to community assembly. The normalized coefficient of each regression model represents the strength of the effect of species abundance on community assembly processes; positive (E) and negative (F) cohesion of BGC‐containing bins and non‐BGC‐containing bins. Cohesion was calculated as the sum of significant positive or negative correlations between species weighted by species abundance, representing the positive and negative co‐occurrence relationships between species in a community, and was used to characterize changes in community connectivity across high temperatures caused by positive/negative species interactions or niche similarities/differences. Statistical significance among groups was assessed using Fisher's LSD test, and differences were indicated by distinct letters (e.g., a, b). (G) The content of BGC‐containing bins was positively correlated with temperature, with colors representing the BGC‐containing bins and each point corresponding to one community at its observed fermentation temperature; the taxon color scheme is shared between (B) and (G).

Positive and negative cohesions were calculated on the basis of species connectedness and abundance via a modified pairwise correlation matrix, and the null model cohesion peaked concurrently with increasing temperature, whereas negative cohesion displayed irregular variations [35, 36]. The PCA results indicated that positive cohesion is a major contributor to community stability (74.95%; Figure S7). All species presented significantly greater positive cohesion (0.303–0.464) than did the community without BGC‐containing bins (0.289–0.461) (Figure 3E), whereas the negative cohesion (−0.215 to −0.152) of the community without BGC‐containing bins was significantly greater than that of all species (−0.224 to −0.154). BGC‐containing bins appeared to increase positive mutualism in the community (Figure 3F). Correlation analysis and multiple linear regression analysis further revealed that BGC‐containing bins demonstrated temperature resilience, maintaining microbial stability under rising temperature conditions (Figure 3G). Therefore, BGC‐containing bins were related to enhance ecological cohesion.

### Correlation between microbial adaptability and the content of BGC‐containing bins at high temperatures

To determine the mechanism by which BGC‐containing bins affect microbiota fitness under high temperatures, co‐occurrence distances based on the Jaccard distance were assessed at each temperature (Figure 4A). The co‐occurrence distances remained stable below 50°C, but showed a significant increase at temperatures from 51.31°C to 61.31°C. Microbial communities responded to increasing temperatures by decreasing α‐diversity and increasing the abundance of BGC‐containing bins. Therefore, we hypothesized that the microbiota developed temperature tolerance by variations in functions and maintained a high degree of interaction at phases above 60°C.

**FIGURE 4:**
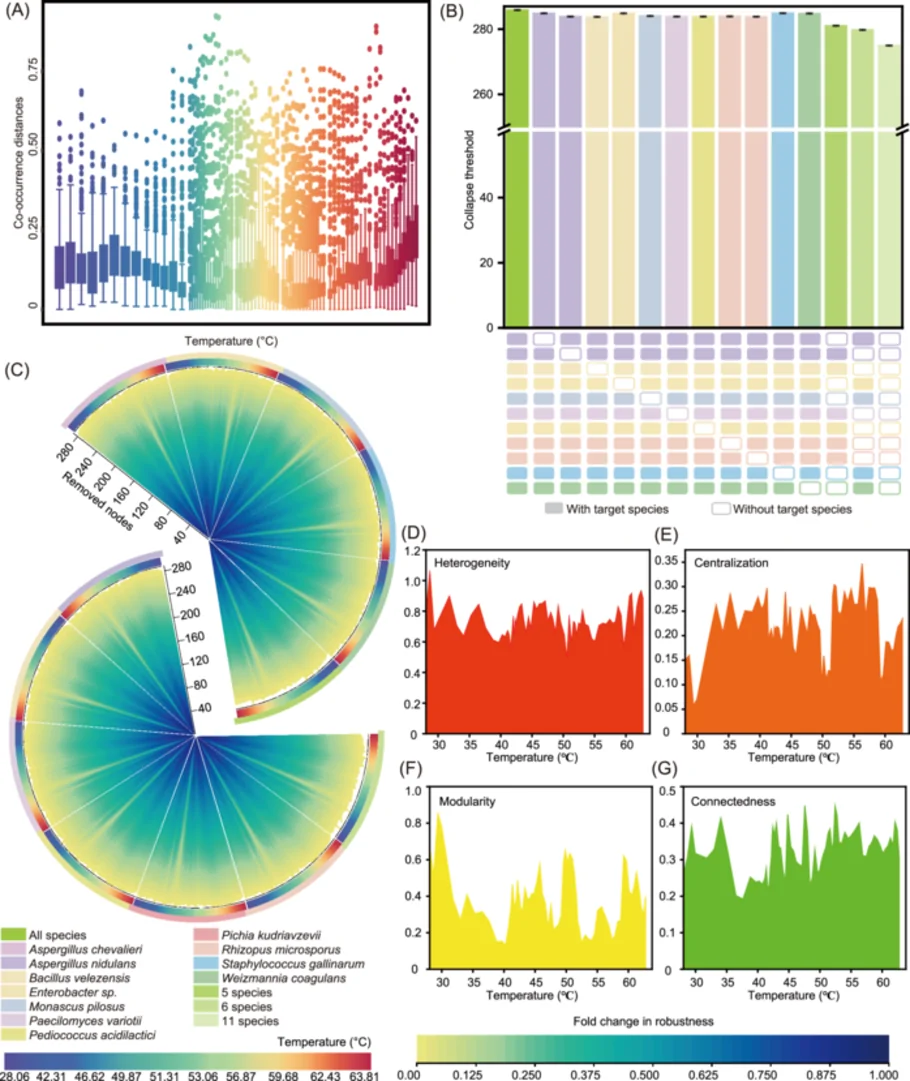
Community stability is limited by the number of biosynthesis gene cluster (BGC)‐containing bins. (A) Microbial co‐occurrence distances during temperature variation. Symbiotic distance was expressed as the Jaccard distance, which was used to identify the survival distance of microorganisms in different communities; the Jaccard dissimilarity reflects minimal survival similarity between communities. (B) Collapse threshold of the microbial community after the removal of each or all 11 BGC‐containing bins. The collapse threshold refers to the maximum number of nodes that can be removed during a random node removal process (*n* = 76) before community robustness decreases to 0. This value represents the minimal number of nodes whose loss collapses the network under random deletion, indicating community collapse. This threshold illustrates the critical point at which the removal of species leads to a significant loss of stability within the community. (C) Effect of removing BGC‐containing bins on community stability; robustness quantifies the network's tolerance to progressive node removals prior to collapse (higher values indicate greater stability). The inner circle denotes the targeted removal of the indicated species/bin, whereas the outer circle denotes temperatures. Temperature colors are consistent with (A), and the species colors are consistent with (B). (D–G) Variations in the characteristics of microbial stability at different temperatures after the absence of BGC‐containing bins.

To assess the significance of BGC‐containing bins in the microbial community, simulations were conducted by individually removing each BGC‐containing bin (Figures 4B,C and S8A). The collapse threshold indicated the highest number of nodes that could be eliminated in a random removal process before the robustness dropped to 0. When all 11 BGC‐containing bins were present, the collapse threshold was 285.868. One‐by‐one removal of *A. nidulans*, *B. velezensis*, *P. variotii*, *P. acidilactici*, *P. kudriavzevii*, and *R. microsporus* decreased the threshold to <283, whereas removal of *A. chevalieri*, *Enterobacter sp*., *M. pilosus*, *S. gallinarum*, and *W. coagulans* yielded thresholds > 284. Consistently, removing the first six and the last five taxa reduced the thresholds to 279.802 and 281.066, respectively, and removing all 11 taxa reduced the collapse threshold to 274.987 (Figure 4B). When the first six species—*A. nidulans*, *B. velezensis*, *P. variotii*, *P. acidilactici*, *P. kudriavzevii*, and *R. microsporus*—were removed, a significant decline in community robustness was observed; the last five species (*A. chevalieri*, *Enterobacter sp*., *M. pilosus*, *S. gallinarum*, and *W. coagulans*), resulted in only a minor decline in robustness to 0.434, which remained significantly greater than the value observed after removing all 11 BGC‐containing bins (0.429) (Figure 4C). These results indicate that the first six taxa contribute disproportionately to network stability; therefore, we refer to them as fundamental species, defined as species that (i) contain BGCs and (ii) are highlighted as key contributors in the iCAMP‐derived phylogenetic bin framework. Here, heterogeneity reflects how unevenly links are distributed among nodes (higher values indicate stronger hub‐structure), centralization indicates whether the network is dominated by a small number of highly connected nodes, modularity describes the degree of partitioning into submodules (often interpreted as buffering perturbations), and connectedness summarizes overall interaction density. In MENs without BGC‐containing bins, heterogeneity, centralization, modularity, and connectedness were all substantially lower than those in the all‐species MENs, indicating weakened interaction structure. Across 55–64°C, the network maintained high topological stability: all topology indices reported here were calculated from the baseline networks without targeted node removal (Figure S8B), heterogeneity fluctuated between 0.760 and 0.866 without a consistent monotonic trend; centralization varied modestly within 0.120–0.200; modularity followed a stage‐like pattern, decreasing from 0.73 (55°C–57°C) to 0.39 (58°C–61°C) and then partially recovering to 0.45 (62°C–64°C); and connectedness declined slightly from 0.050 to 0.029 at the highest temperatures (Figure 4D–G). Together, these results support that the microbial network preserves robust topological stability under high‐temperature stress, and that fundamental species help sustain interaction density and buffer stability loss during the extreme‐temperature phase (Table S5). Co‐occurrence analyses indicated that BGC‐containing bins maintained higher interaction density at extreme temperatures, buffering the loss of diversity and facilitating rapid recovery after disturbance. Their absence led to fragmentation of interaction networks, confirming their role as stability anchors in thermal‐stressed communities.

### The role of the biomass of BGC‐containing bins in maintaining microbial stability

BGC‐containing bins maintained microbiota abundance at high temperatures, but it remains unclear whether these characteristics persisted below 50°C. Therefore, the effects of secondary metabolites on microbial mutualism and microbial survival at all temperatures were analyzed. To distinguish between the effects of BGCs and BGC‐containing bins, a Procrustes analysis was used to assess data consistency and similarity, which revealed that the contribution of BGCs to microbial stability also arose from BGC‐containing bins (Figures 5A and S9A). Further analysis of matrix correlations revealed that 90.3% (149/165) of the BGC‐containing bins significantly affected the BGC content, underscoring the importance of BGC‐containing bins (Figure 5B). We also evaluated correlations between temperature and BGC‐containing bins and other species, and not all BGC‐containing bins exhibited significant temperature tolerance (Figure S9B). In the isolate‐based Synthetic Communities (SynComs) experiment, the inoculation ratio refers to the proportion of fundamental species: non‐BGC‐containing species. During the 36°C cultivation, the total biomass of communities with an inoculation ratio of 1:0 reached the highest value (9.44 Lg copies/g), whereas the 0:1 ratio group presented a biomass of 6.29 Lg copies/g (biomass has increased by more than 50.08%). At 47°C, the 1:3 inoculation ratio resulted in the highest biomass (10.82 Lg copies/g), whereas the 0:1 ratio decreased sharply to 3.43 Lg copies/g (increased by more than 215.45%). For 57°C cultivation, the 1:0 ratio group maintained dominance with 11.08 Lg copies/g, whereas the 0:1 ratio group demonstrated 3.57 Lg copies/g (increased by more than 210.36%). Under extreme 61°C conditions, the 1:3 ratio group had the highest biomass (5.32 Lg copies/g), which contrasted sharply with the 0:1 ratio group (3.50 Lg copies/g, increased by more than 52.00%) (Figure 5C). This suggested that microbial stability relied on the existence of fundamental species rather than on the resilience of all species. Thus, we selected six fundamental species and six other species (species that neither contributed to community structure nor contained BGCs) to perform validation experiments. Experiments across four temperature gradients confirmed that BGC‐containing bins increased the overall temperature tolerance of the microbiota. According to these experimental results, the presence of BGC‐containing bins promoted an overall increase in microbial abundance and mitigated the decline of other species under high‐temperature conditions, thereby enhancing ecological cohesion and contributing to microbial stability under thermal stress.

**FIGURE 5:**
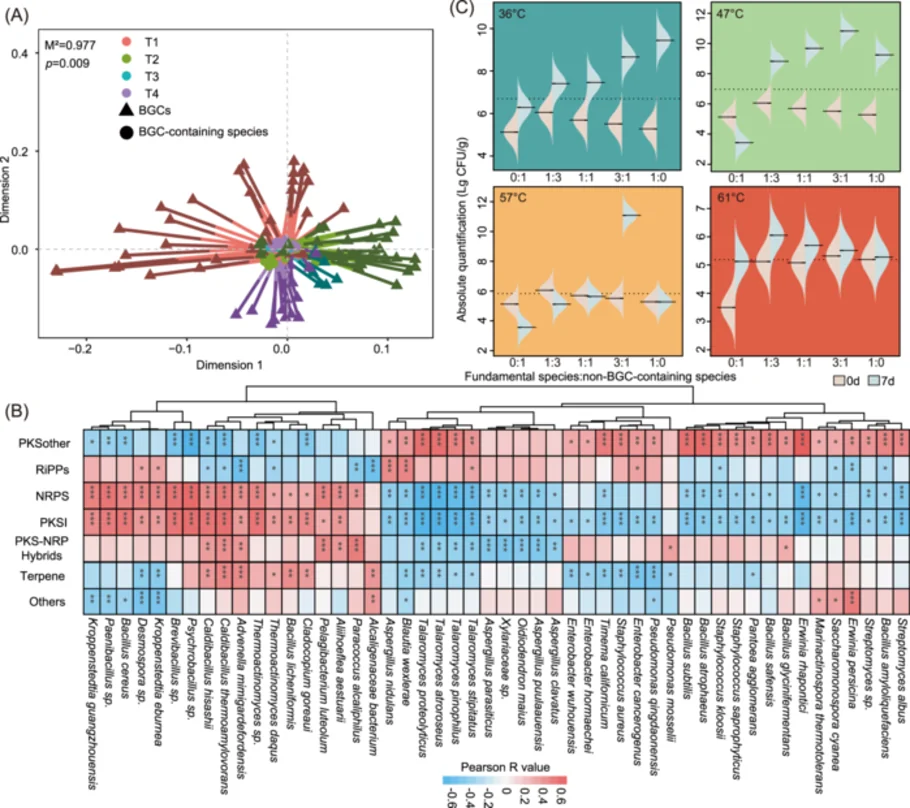
BGC‐containing bins regulate secondary metabolic genes, community stability, and community species abundance in communities. (A) Procrustes analysis was used to evaluate the degree of correlation between BGC‐containing bins and secondary metabolic genes. Triangles represent BGC data, and circles represent the abundance data of BGC‐containing bins. M^2^ represents the sum of squares of the deviation between the coordinates of the two data points and the corresponding points after transformation. T1: 36°C, T2: 47°C, T3: 57°C, T4: 61°C. (B) Correlation test between bins containing the BGC matrix and secondary metabolic gene matrix. (C) Changes in the contents of BGC‐containing bins and non‐BGC‐containing bins in the co‐culture experiment of the synthetic community (SynComs), which is performed with triplicate biological replicates for each experimental group. The presence of BGC‐containing bins increased the content of total community species. *p < 0.05, **p < 0.01, ***p < 0.001.

## 3 DISCUSSION

This study highlighted the crucial role of temperature as both a “destroyer” and a “reconstructor” in shaping microbial community assembly, especially under high‐temperature stress. During the construction of the molecular ecological networks, BGC‐containing bins dominated the assembly process by fostering microbial interactions and stabilizing the communities in high‐temperature environments. On the basis of our findings, the presence of BGC‐containing bins (fundamental species) improved microbial stability by dynamically responding to community assembly and promoting a more stable microbial community. Our validation experiments confirmed that BGC‐containing bins significantly increased microbial stability, ensuring community resilience and functional persistence under extreme thermal stress. Our findings underscore the central role of BGCs in sustaining stability and enabling adaptation to thermal stress. Interestingly, in addition to the high‐temperature features emphasized here, the lower‐temperature stage also shows pronounced phase‐structured patterns. This motivates interpreting the temperature trajectory in a stage‐aware manner, where sub‐range analyses can reveal distinct ecological regimes across fermentation.

In our study, we proposed that BGC‐containing bins maintained microbial community stability through their environmental tolerance and classified them as fundamental species [37, 38]. Although summer exhibited the highest ambient temperatures, the fermentation temperature did not peak accordingly (Figure S1), implying microbial modulation of metabolic heat to maintain stability of fermentation temperature. This autoregulation highlights the ecological relevance for natural microbiota facing fluctuating thermal conditions. This definition extends the keystone concept beyond abundance, emphasizing niche occupation, resource use, and adaptability to environmental change [39]. This study identified six fundamental species—*A. nidulans*, *B. velezensis*, *P. variotii*, *P. acidilactici*, *P. kudriavzevii*, and *R. microsporus*—that drove microbial community assembly through multidimensional synergistic mechanisms. As previously reported, *B. velezensis* mediates dual regulation via metabolite secretion for functional synergy and pathogen suppression [40]; *P. acidilactici* suppresses competing microbiota via environmental acidification [5]; *R. microsporus* facilitates symbiotic colonization by degrading organic substrates [41]; and *A. nidulans* likely increases environmental adaptability through stress‐resistant metabolites [42]. Together, they stabilized community function via network remodeling, competitive partitioning, and coordinated stress responses. Secondary metabolites played crucial roles in mediating microbial interactions, contributing to various forms of interspecies interactions. For example, microorganisms often secrete toxic secondary metabolites to confer a competitive advantage over other species in co‐culture [43, 44, 45]. In addition, secondary metabolites can also influence transcriptional regulation, virulence, motility, biofilm formation, and consequently community assembly at sub‐inhibitory concentration [46, 47, 48, 49].

BGC‐containing bins exhibited transient decreases in abundance during the critical temperature transition from 53°C to 55°C. Therefore, they might have temperature tolerance, and these bins with strong environmental tolerance can accelerate the recovery process of the community by rapidly restoring the population and re‐establishing the microbial community after the community is disturbed or destroyed [50, 51, 52, 53]. BGC‐containing bins facilitated mutualism through their high‐temperature tolerance, thereby increasing the stability of the microbial community. In environments exposed to high temperatures, microorganisms have the capacity to bolster the heat tolerance of algae, fostering a mutually beneficial symbiosis that aids algae in enduring extreme conditions [54]. This interplay not only bolsters the viability of individual species but also contributes to the overall stability of the microbial community [55, 56]. Consequently, temperature tolerance has emerged as a pivotal factor underpinning microbial mutualism and stability, offering a fresh perspective on interspecies dynamics.

The removal of fundamental species led to an imbalance within the microbiota, accompanied by a significant decrease in the intensity of mutualism [57, 58]. Fundamental species played pivotal roles in sustaining microbial stability and functional diversity, and their absence disrupted the delicate equilibrium within these communities. To assess the impact of species loss on community structure, we proposed a conceptual framework designated the steady‐state coefficient. We argued that under external environmental stress, the stability of microbial communities was reflected in feedback regulation within the community. Negative feedback stabilized ecosystems by inhibiting and dampening initial changes, whereas positive feedback accelerated these changes, driving the system away from stability [59]. Therefore, the steady‐state coefficient that describes microbial stability should focus on negative feedback regulation: the greater the connectivity between nodes is, the stronger the negative interaction is, and the lower the network clustering is, the greater the stability of the community is. The interactions within species, including nutrient competition [60], symbiotic relationships [61, 62], and signaling mechanisms [63], fundamentally relied on the existence of these pivotal fundamental species [64]. However, once they began to decline or were completely removed, the MENs became fragile, resulting in a marked impairment of overall function [65, 66]. Specifically, microbial diversity decreased [67], and the population of beneficial microbiota dwindled [66, 68]. These transformations further exacerbated the degree of microbial imbalance that had already been established.

The adaptation of the microbial community to rapid environmental variation is a key issue in the context of climate change. As temperatures rise, understanding how complex microbiota systems (e.g., spontaneous fermentation) adapt is crucial, as they cannot achieve the classification culture of all strains. Focusing on fundamental species can help regulate microbial community assembly and mutualism. This study provided insights into the role of fundamental species in microbial mutualism in high‐temperature environments, highlighting the important role of BGC‐containing bins in maintaining microbial stability. In contrast to their typical ‘competitor’ label, BGC‐containing bins maintain microbial stability under high‐temperature conditions. Therefore, further validation of these findings at the metabolite level and in higher‐order interactions is needed, particularly to understand the implications of these dynamics in the face of ongoing environmental stress.

## 4 CONCLUSION

In conclusion, by integrating iCAMP‐based phylogenetic‐bin assembly inference with molecular ecological network analyses and isolate‐based SynCom validation, we show that microbial community organization during *Daqu* spontaneous fermentation is tightly associated with temperature and displays clear stage‐specific dynamics across the 20–65°C trajectory. BGC‐containing phylogenetic bins and the subset of taxa defined here as fundamental species were consistently linked to higher ecological cohesion and stability‐related network properties under thermal stress, and removal simulations highlighted their disproportionate contribution to network robustness and collapse threshold. Notably, SynCom experiments further supported the functional relevance of these taxa by demonstrating temperature‐dependent biomass advantages under specific inoculation ratios. Together, our results nominate BGC‐associated fundamental species as key stability‐linked components of thermally stressed fermentation ecosystems and provide a framework for dissecting how phylogenetic structure and secondary metabolic potential relate to community resilience under warming conditions.

## 5 METHODS

### Sample collection and thermometry

Samples were taken from spontaneous fermentation (between September 2020 and June 2021) at a well‐known Chinese liquor distillery located along the Chishui River. A consistent fermentation method was used for fermentation over 4 seasons as previously reported [69]. Sampling was carried out on Days 1, 3, 7, 9, 11, 15, and 39, depending on the temperature variation. To minimize heterogeneity, each sample was prepared as a composite by mixing five subsamples from the same pile (four edges and one center); these subsamples were combined into one sample and were not treated as independent replicates. Three fermentation chambers were sampled as biological replicates at each time point, and sampling was performed at seven time points across four seasonal fermentation batches. Finally, a total of 84 samples were collected, immediately frozen in liquid nitrogen, and stored at −80°C for further analysis. Fermentation temperature was continuously monitored (Figure S1), and temperature variation among biological replicates was small (coefficient of variation, CV < 10%).

### Metagenomic sequencing and data analysis

#### Total DNA extraction

The acquired DNA for metagenomic sequencing was extracted with the Omega Soil DNA Kit (Omega Biotek, Norcross, GA) according to the manufacturer's specifications. The purity and concentration of the DNA were tested with a Qubit 2.0 (Thermo Fisher Scientific) and Nanodrop One (Thermo Fisher Scientific), respectively. After a quality check and quantification, DNA library construction was accomplished following the Illumina TruSeq DNA Sample Preparation Guide (Illumina). The shotgun sequencing of the DNA library was performed on the Illumina NovaSeq. 6000 platform (Illumina).

#### Metagenomic analysis

The raw reads of each metagenomic sequence dataset were filtered to remove adapter sequences and low‐quality reads (quality score < 20) with Trim Galore (v 0.5.0) (http://www.bioinformatics.babraham.ac.uk/projects/trim_galore/) with default parameters [70]. The quality of the reads was checked with FastQC (v 0.11.8) (https://www.bioin formatics. babraham. ac. uk/projects/fastqc/). A total of 396.7 Gb of clean reads were retained. The clean reads of each sample were used for assembly with MEGAHIT (v 1.1.3) with default parameters [71]. The clean reads from the same study were subsequently mixed into one fastq file (for pair‐end sequencing data, upstream and downstream sequencing data files were mixed separately). For the obtained contigs, CD‐HIT (v 4.8.1) was used to remove redundancy with the following parameters: ‐c 0.95 ‐M 640000 ‐T 24 ‐n 5 ‐d 0 ‐aS 0.9 ‐g 1 ‐sc 1 ‐sf 1 [72]. The redundant contigs were used for predicting genes and protein sequences by Prodigal (v 2.6.3) [73], and then, we used EukRep (v 0.6.7) to distinguish the eukaryotic and prokaryotic sequences. The taxonomic annotations of nonredundant genes were annotated with DIAMOND (v 0.9.35.136) with a DIAMOND database built from the National Center for Biotechnology Information (NCBI) NR database [74]. The functional annotations of nonredundant genes were performed with eggNOG‐mapper software (v 2.1.12) with the eggNOG database (v 5.0.2). We quantified genes with Bowtie2 (v 2.2.5) [75], and the abundances of all the genes were normalized to the number of fragments per kilobase per million mapped reads (Reads Per Kilobase Million, RPKM) [76].

#### Biosynthesis gene cluster analysis

BGCs for eukaryotic and prokaryotic sequences were predicted with antiSMASH (v 6.0) fungal and bacterial versions with the following parameters [77]: ‐‐taxon bacteria ‐‐output‐dir ‐‐genefinding‐tool prodigal ‐‐cb‐knownclusters ‐c 4 ‐‐cc‐mibig ‐‐cb‐general ‐‐cb‐subclusters‐fullhmmer.

### Molecular ecological network analysis

MEN was constructed using taxa abundance profiles on Molecular Ecological Network Analysis (MENA, http://ieg4.rccc.ou.edu/mena/register.cgi). To examine temperature‐dependent interaction patterns, we applied a sliding‐window approach in which every eight samples with adjacent temperature values were grouped together, and their average taxa abundance was used as the input for network construction. This procedure generated 76 MENs in total. To ensure the reliability of the constructed network, only the RPKM values of species higher than 1 were included for further correlation calculations. The network was constructed on the basis of the Pearson correlations of log‐transformed species RPKM values. All interaction thresholds were calculated by random matrix theory (RMT) to filter node associations [78]. The topological indices of the constructed network were calculated on the MENAP interface (http://ieg4.rccc.ou.edu/mena/register.cgi). All networks were visualized with WGCNA (v 1.72–1), igraph (v 1.3.0), dplyr (v 1.1.3), Hmisc (v 4.7–0), and reshape2 (v 1.4.4) packages in RStudio.

In MEN construction, not all detected species are retained as network nodes after abundance filtering and RMT‐based thresholding; therefore, the number of nodes is typically smaller than the number of visible species within a window. We quantified MEN stability using node persistence, defined as nodes/visible species for each of the 76 MENs. To evaluate important nodes for maintaining network stability, we employed robustness [13] to characterize the resilience of MENs, which measures the network's resilience to node loss, either through random removal or targeted deletion of specific nodes. To simulate random species loss, a fixed proportion of nodes was removed, independent of taxa. To assess the contribution of foundational species, targeted node removal simulations were conducted in two steps: nodes containing these species were first eliminated, followed by random removals of other nodes. This approach helps verify the significance of these foundational species in preserving structural and functional integrity under disturbance. The entire analysis was implemented in RStudio, utilizing the “foreach” and “doParallel” packages to enable efficient parallel computation throughout the simulation process.

### Isolation of strains and co‐culture experiment

For strain isolation, to separate the species in the fermentation system, the sample was mixed with PBS at a ratio of 1:10 for gradient dilution. The dilution series (10^−4^–10^−6^) was selected, bacteria were isolated with LB medium (with amphotericin added), yeast was isolated with WL medium (with penicillin‐streptomycin and chloramphenicol added), and mold was isolated with Rose Bengal medium.

We established five sets of synthetic communities (SynComs) in a solid‐state system for co‐culture. In the first series, six fundamental species were co‐cultured at equal cell concentrations. In the second series, the same six fundamental species and six non‐BGC‐containing species were co‐cultured at a 3:1 inoculation ratio. In the third and fourth series, the inoculation ratio of fundamental to non‐BGC‐containing species was adjusted to 1:1 and 1:3, respectively. In the fifth series, six non‐BGC‐containing species were co‐cultured at equal cell concentrations. The total cell concentration of each of the above five groups was 1 × 10^6^ CFU/g, within each group, all strains classified as fundamental species were inoculated at equal cell concentrations, and all strains classified as non‐BGC‐containing species were inoculated at equal cell concentrations. To prepare the culture medium, wheat (500 g) and water (750 mL) were mixed in a 3 L beaker; the mixture was maintained for 6 h at 4°C and autoclaved at 105°C for 45 min. After cooling, the wheat solid culture medium was acquired. We added the different SynComs to the wheat medium and cultured for 7 days at four different temperatures (Figure S10). Fermented samples were collected at Days 0 and 7 of fermentation, and three biological replicates were used per culture. All the samples were stored at −80°C for qPCR analysis.

### Statistical analysis

Unless otherwise stated, community‐level ordination, α/β‐diversity analyses, and MEN construction were performed using species‐level taxonomic abundance profiles normalized as RPKM. In contrast, functional analyses (including BGC‐related analyses) were based on gene/BGC annotations and their corresponding abundances or counts. The α‐diversity of bacteria, fungi, and total species, and the co‐occurrence distances were analyzed with the “picante” package. NMDS and ANOSIM (analysis of similarities) were used to identify the significant differences among groups with the “vegan” package (v 2.7–2). Differences were considered statistically significant at *p* < 0.05 (two‐tailed). Community assembly processed and phylogenetic groups were analyzed via iCAMP analysis [15]. In iCAMP analysis, bin (phylogenetic group) refers to a group formed by clustering ASV based on their phylogenetic relationships. It is computed in two main steps: (1) calculating phylogenetic distances: computing pairwise evolutionary distances between all species based on the phylogenetic tree; (2) clustering according to thresholds: setting a phylogenetic distance threshold (ds = 0.2) and a minimum bin size (bin.size.limit = 24), then aggregating species whose pairwise phylogenetic distances are less than or equal to the threshold into a single bin, ensuring each bin contains a sufficient number of species for subsequent statistical analysis. Here, “bin” refers to an iCAMP phylogenetic bin (phylogenetic group), and “BGC‐containing bins” denotes phylogenetic bins that include BGC‐containing taxa based on taxonomic profiles and BGC annotations; MAG binning was not performed.

To quantify how different bins contribute to community assembly processes, we conducted linear regression analyses using four predictor variables: bins_bacteria, bins_fungi, bacteria containing BGCs, and fungi containing BGCs. For each ecological process, the relative contribution of the process was used as the response variable. All predictor variables were z‐score normalized prior to model fitting to ensure comparability of regression coefficients. Independent regression models were fitted for each process using the standardized abundance matrix generated in the main analysis pipeline. The normalized regression coefficient (*β*) was extracted and interpreted as the effect strength of each bin category on community assembly. All input matrices and resulting coefficient tables were exported within the analysis script to ensure reproducibility and transparency.

Cohesion is a metric that quantifies microbial community connectivity by separately measuring the strength of positive and negative species co‐occurrence patterns. Community dissimilarity was calculated as the Aitchison distance and the Jaccard distance from the nonrarefied dataset, and the distance between sample pairs was calculated as the Euclidean distance, which was calculated in R with the “compositions” package. The correlation between BGC‐containing bins and secondary metabolic genes was analyzed by Procrustes analysis with the “vegan” package (v 2.7–2) in RStudio.

## AUTHOR CONTRIBUTIONS

**Shibo Ban**: Conceptualization; methodology; investigation; data curation; formal analysis; visualization; writing—original draft and editing. **Binghao He**: Data curation; investigation; validation. **Yang Song**: Methodology; software; writing—review and editing. **Xin Li An**: Methodology; formal analysis; writing—review and editing. **Yan Xu**: Project administration; funding acquisition; supervision. **Qun Wu**: Conceptualization; project administration, funding acquisition; supervision; writing—review and editing. **Yu Shi**: Conceptualization; methodology; supervision; writing—review and editing. All authors have read the final manuscript and approved it for publication.

## CONFLICT OF INTEREST STATEMENT

The authors declare no conflicts of interest.

## ETHICS STATEMENT

No animals or humans were involved in this study.

## Supporting information


**Figure S1.** Temperature variations in the 84 fermentation samples.
**Figure S2.** Abundance of microorganisms at different seasons.
**Figure S3.** The microbial α‐diversity among fermentation days and temperatures.
**Figure S4.** Visualization of constructed molecular ecological networks (MENs) across temperature from 28.06 °C to 63.81 °C.
**Figure S5.** Correlation between temperature and microbial community attributes.
**Figure S6.** Distribution of microbial functions based on metabolism of COG categories.
**Figure S7.** Positive/negative cohesion and species distribution.
**Figure S8.** Robustness distribution in a network with different temperature interactions.
**Figure S9.** Correlation analysis of BGC‐containing species and temperature during fermentation.
**Figure S10.** Workflow of the effects of BGC‐containing bins on the stability of biomass.

**Table S1.** Dimensionality reduction analysis of data.
**Table S2.** Proportion of bacterial and fungal community assembly processes.
**Table S3.** Nodes' properties of the MENs.
**Table S4.** The amount of BGC contained in species.
**Table S5.** Source data for overall network.

## Data Availability

The raw sequence data were deposited in the NCBI Sequence Read Archive (SRA) databases under the accession numbers PRJNA1290789 (https://www.ncbi.nlm.nih.gov/bioproject/PRJNA1290789). The data and scripts used are saved in GitHub (https://github.com/Lucas-Ban/Daqu-community-analysis.git). Supplementary materials (figures, tables, graphical abstract, slides, videos, Chinese translated version, and update materials) may be found in the online DOI or iMeta Science http://www.imeta.science/imetaomics/.
