# Supplementary material for: Biosynthesis gene cluster‐containing species drive microbial community stability under thermal stress through enhanced ecological cohesion
Source: IMetaOmics. 2026 Feb 15;3(1):e70082. doi: 10.1002/imo2.70082 (PMC13674450; doi:10.1002/imo2.70082)
Supplement: Supplementary file 1 [file IMO2-3-e70082-s002.docx]

Supporting information to

**Biosynthesis gene cluster-containing species drive microbial community stability under thermal stress through enhanced ecological cohesion**

**Running title:** BGC-containing species contribute microbial stability under thermal stress

Shibo Ban^1,2,3^, Binghao He^1^, Yang Song^4^, Xin Li An^5^, Yan Xu^1^, Qun Wu^1*^, Yu Shi^5*^

^1^ Key Laboratory of Industrial Biotechnology of Ministry of Education, State Key Laboratory of Food Science and Resources, School of Biotechnology, Jiangnan University, Wuxi 214122, China

^2^ Kweichow Moutai Co, Ltd, Zunyi 564501, China

^3^ Guizhou Key Laboratory of Microbial Resources Exploration in Fermentation industry，Kweichow Moutai Group, Zunyi 564501,China

^4^ Plant-Microbe Interactions, Institute of Environmental Biology, Department of Biology, Science4Life, Utrecht University, Utrecht 3584 CH, the Netherlands

^5^ State Key Laboratory of Crop Stress Adaptation and Improvement, School of Life Sciences, Henan University, Kaifeng 475004, China

*Correspondence: [wuq@jiangnan.edu.cn](mailto:wuq@jiangnan.edu.cn) (Qun Wu), [yshi@henu.edu.cn](mailto:yshi@henu.edu.cn) (Yu Shi)

**Figure S1 Temperature variations in the 84 fermentation samples.** The temperature profile typically exhibits three distinct rising phases. The first rise occurs rapidly, reaching over 60°C within approximately 4 d and sustaining for 3–4 d. Subsequently, the temperature decreases to around 40°C for about 2 d before entering the second rising phase, where it increases again to around 55°C (approximately 50°C in summer) for another 3–4 d. Following a gradual cooldown, a third temperature rise then occurs after roughly 3 d, days, with temperatures exceeding 50°C, before eventually decreasing to ambient levels during the remaining fermentation remaining fermentation period.

**Figure S2 Abundance of microorganisms at different seasons.** Only those genera and metabolites that their average RPKM (Reads Per Kilobase of transcript, per Million mapped reads) greater than 20 in all samples are indicated. Genera with RPKM less than 20 are combined and shown in the “Others” category. Error bars indicate the standard deviation (SD) of three biological replicates (*n* = 3).

**Figure S3 The microbial α-diversity among fermentation days and temperatures.** Simpson (A and B) and Richness (C and D) displayed as time-series and temperature gradients, horizontal lines indicate the average value. ANOSIM is used to calculate R and *p* values. T1: 25–30 °C; T2: 30–35 °C; T3: 35–40 °C; T4: 40–45 °C; T5: 45–50 °C; T6: 50–55 °C; T7: 55–60 °C; T8: 60–65 °C.

**Figure S4 Visualization of constructed molecular ecological networks (MENs) across temperature from 28.06 °C to 63.81 °C.** Large modules are shown in different colors, and smaller modules are shown in grey.

**Figure S5 Correlation between temperature and microbial community attributes.** Microbial community attributes include (A) species abundance, (B) β-distance, (C) α-diversity, and (D) node persistence. The shaded areas represent the 95% confidence intervals for the fitted lines. Pearson correlation coefficients (R) and two-tailed *p*-values were calculated using standard Pearson correlation tests.

**Figure S6 Distribution of microbial functions based on metabolism of COG categories.** [C] Energy production and conversion, [E] Amino acid transport and metabolism, [F] Nucleotide transport and metabolism, [G] Carbohydrate transport and metabolism, [H] Coenzyme transport and metabolism, [I] Lipid transport and metabolism, [P] Inorganic ion transport and metabolism, [Q] Secondary metabolites biosynthesis, transport and catabolism. Pearson correlation coefficients (R) and two-tailed *p*-values were calculated using standard Pearson correlation tests.

**Figure S7 Positive/negative cohesion and species distribution.** Principal component analysis (PCA) indicates that positive cohesion is the first principal component, with the community explaining 74.95% of the variance in positive cohesion in the presence of bins containing BGCs.

**Figure S8 Robustness distribution in a network with different temperature interactions.** (A) robustness is defined as the resistance of the network to node loss after random or target node removal. For simulated random species removal, a certain proportion of nodes are randomly removed. For simulated target species removal, a certain number of key nodes are removed. (B) Network coefficients at different temperatures.

**Figure S9 Correlation analysis of BGC-containing species and temperature during fermentation.** (A) Correlation between temperature and both the abundance of BGC-containing species and the number of BGC-containing species in fermentation. (B) Correlation between temperature and the abundance of BGC-containing species. Pearson correlation coefficients (R) and two-tailed *p*-values were calculated using standard Pearson correlation tests.

**Figure S10 Workflow of the effects of BGC-containing bins on the stability of biomass.** (A) 12 strains were selected for BGC-containing bins (SM) and non-BGC-containing species (NSM), with yellow boxes representing the BGC-containing bins and green boxes representing the other species. Synthetic community (SynCom) was constructed with 1:1 content mixing; (B) The ratio of BGC-containing bins to other species was 4 gradients, and co-culture was carried out under 4 temperature gradients (see Figure 2 for temperature selection). A total of 20 groups in experiment (*n* = 3 each co-culture); (C) SynCom was inoculated in wheat solid medium and cultured in a temperature incubator. For the preparation method of culture medium, see Materials and Methods; (D) The metagenome was extracted from the co-culture samples and qPCR was performed to quantify the content of BGC-containing bins and other species.
